# Author Correction: Competitive endogenous RNA is an intrinsic component of EMT regulatory circuits and modulates EMT

**DOI:** 10.1038/s41467-019-13370-4

**Published:** 2019-11-20

**Authors:** Yuwei Liu, Mengzhu Xue, Shaowei Du, Wanwan Feng, Ke Zhang, Liwen Zhang, Haiyue Liu, Guoyi Jia, Lingshuang Wu, Xin Hu, Luonan Chen, Peng Wang

**Affiliations:** 1 Laboratory of Systems Biology, Shanghai Advanced Research Institute, Chinese Academy of Sciences, 200031 Shanghai, China; 2 University of Chinese Academy of Sciences, 200031 Shanghai, China; 3 School of Life Sciences, Shanghai University, 200031 Shanghai, China; 4 Bio-Med Big Data Center, CAS Key Laboratory of Computational Biology, CAS-MPG Partner Institute for Computational Biology, Shanghai Institute of Nutrition and Health, Shanghai Institutes for Biological Sciences, Chinese Academy of Sciences, 200031 Shanghai, China; 5 Key Laboratory of Systems Biology, Center for Excellence in Molecular Cell Science, Institute of Biochemistry and Cell Biology, Shanghai Institutes for Biological Sciences, Chinese Academy of Sciences, 200031 Shanghai, China; 6 Shanghai Institute of Materia Medica, Chinese Academy of Sciences, 200031 Shanghai, China; 7 School of Life Science and Technology, ShanghaiTech University, 201210 Shanghai, China; 8 Department of Breast Surgery, Key Laboratory of Breast Cancer in Shanghai, Fudan University Shanghai Cancer Center, Fudan University, 200032 Shanghai, China; 9 Precision Cancer Medicine Center, Fudan University Shanghai Cancer Center, 200032 Shanghai, China; 10 Center for Excellence in Animal Evolution and Genetics, Chinese Academy of Sciences, 650223 Kunming, China; 11 Research Center for Brain Science and Brain-Inspired Intelligence, 201210 Shanghai, China

**Keywords:** Cancer, Gene regulatory networks, miRNAs, Dynamic networks

Correction to: *Nature Communications* 10.1038/s41467-019-09649-1, published online 09 April 2019.

The originally published version of this Article contained an error in Figure 3. In panels D and G, the western blot lanes in the order left-to-right ‘siCtrl + ctrl UTR’, ‘siTGFBI + ctrl UTR’ and ‘siTGFBI – TGFBI UTR’ were incorrectly labelled in the order ‘siTGFBI – TGFBI UTR’, siTGFBI + ctrl UTR’ and ‘siCtrl + ctrl UTR’. This error has now been corrected in both the PDF and HTML versions of the Article.

