# Supplementary material for: Competitive endogenous RNA is an intrinsic component of EMT regulatory circuits and modulates EMT
Source: Nat Commun. 2019 Apr 9;10:1637. doi: 10.1038/s41467-019-09649-1 (PMC6456586; doi:10.1038/s41467-019-09649-1)
Supplement: Supplementary file 1 — Supplementary Information [file 41467_2019_9649_MOESM1_ESM.pdf]

Competitive endogenous RNA is an intrinsic component of EMT regulatory circuits and modulates EMT

Liu et al.

Supplementary Figures

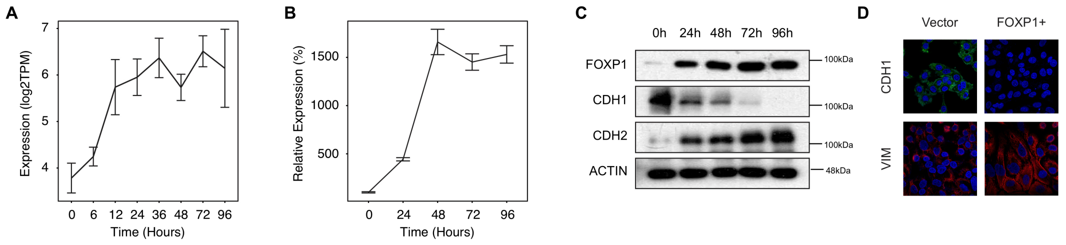

Supplementary Figure 1. **FOXP1 is a critical inducer of EMT in A549 cells.**

(A) RNA-seq data showing monotonic upregulation of FOXP1 during TGF-β-induced EMT in A549 cells. (B) Same as (A) using qRT-PCR. (C) Same as (A) for immunoblotting analysis. (D) Confocal microscopy images showing the upregulation of VIM protein and the downregulation of CDH1 protein in A549 cells overexpressing FOXP1. n = 3; error bars indicate the means ± s.d.

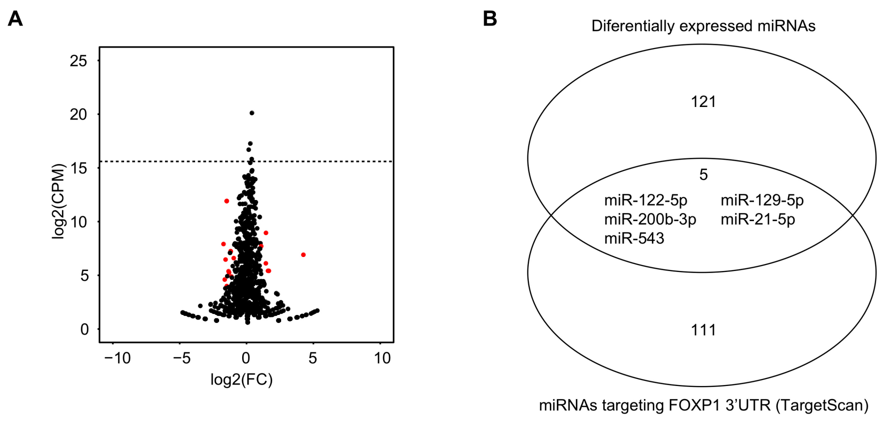

Supplementary Figure 2. **Sequencing analysis indicates that miR-21 is the key miRNA regulating EMT in A549 cells.**

(A) Volcano plot showing the differential expression of miRNAs at 24 h into TGF-β-induced EMT in A549 cells. The red dots represent miRNAs with a differential expression FDR < 0.05 and absolute log2-fold change > 1. The horizontal dotted line represents the log2(CPM) corresponding to 100 copies/cell. (B) Venn diagram showing the overlap between the miRNAs that were differentially expressed at 96 h into TGF-β-induced EMT in A549 cells and the miRNAs predicted by targetScan to regulate FOXP1.

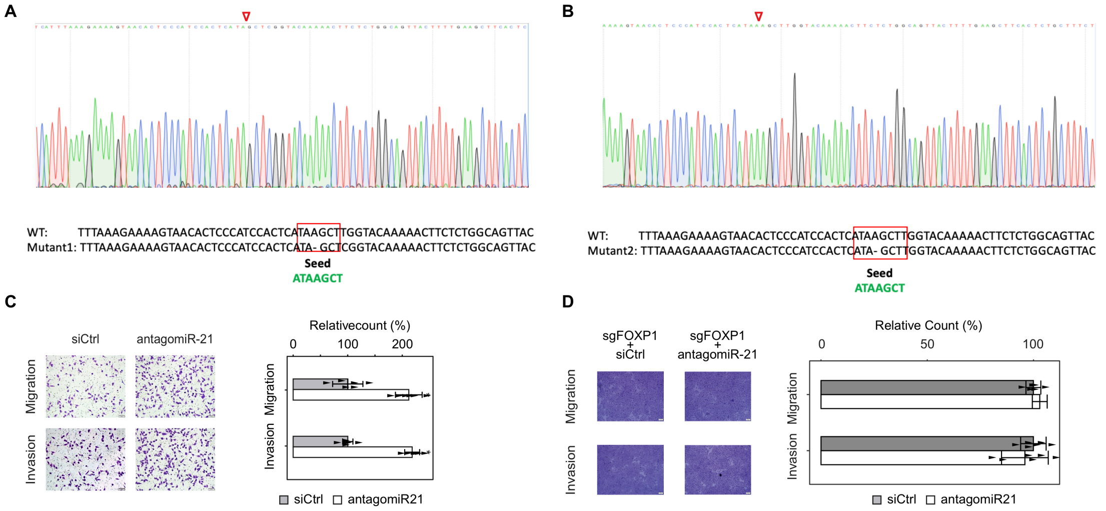

Supplementary Figure 3. **Sequencing traces of the CRISPR/Cas9 edited miR-21 binding site in FOXP1 3'UTR.**

(A) and (B) showing traces of genomic sequence surrounding the miR-21 binding site in FOXP1 3'UTR (upper panels) and corresponding primary sequences (lower panels) for the two alleles of a representative clone. Red boxes highlight the mutated miR-21 binding sites. Red triangles mark the mutation sites in sequencing traces. (C) A549 cells undergoing TGF-β-induced EMT were treated with an antagomiR targeting miR-21 and subjected to a migration assay (upper panel) and invasion assay (lower panel). Scale bars: 100 µm. The migrated or invaded cells were quantified (bar charts). n = 6. (D) A549 cells whose miR-21 binding site in FOXP1 has been mutated by CRISPR/Cas9 undergoing TGF-β-induced EMT were treated with an antagomiR targeting miR-21 and subjected to a migration assay (upper panel) and invasion assay (lower panel). Scale bars: 20 µm. The migrated or invaded cells were quantified (bar charts). n = 6. error bars indicate the means ± s.d. * $p < 0.01$, determined using a two-tailed Student's *t*-test.

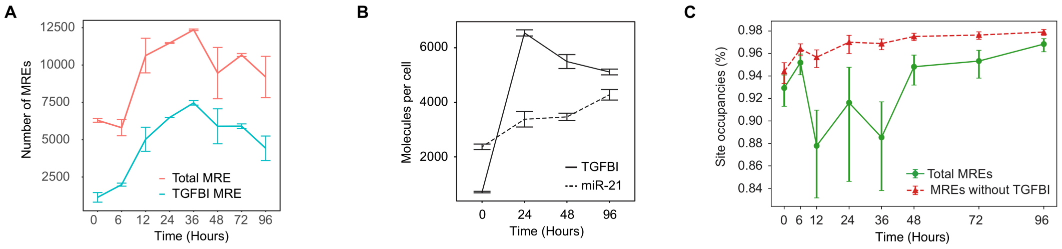

Supplementary Figure 4. **Dynamically induced mRNAs as ceRNA regulators of EMT in A549 cells.**

(A) Graph showing the number of miR-21 MREs extrapolated from RNA-seq data during TGF-β-induced EMT in A549 cells using pictar-based predictions. (B) Absolute quantification of the numbers of TGFBI mRNA and miR-21 transcripts during TGF-β-induced EMT in A549 cells. (C) Graph showing the dynamics of modeled 8-mer miR-21 binding site occupancy during TGF-β-induced EMT in A549 cells using pictar-based predictions. n = 3; error bars indicate the means ± s.d.

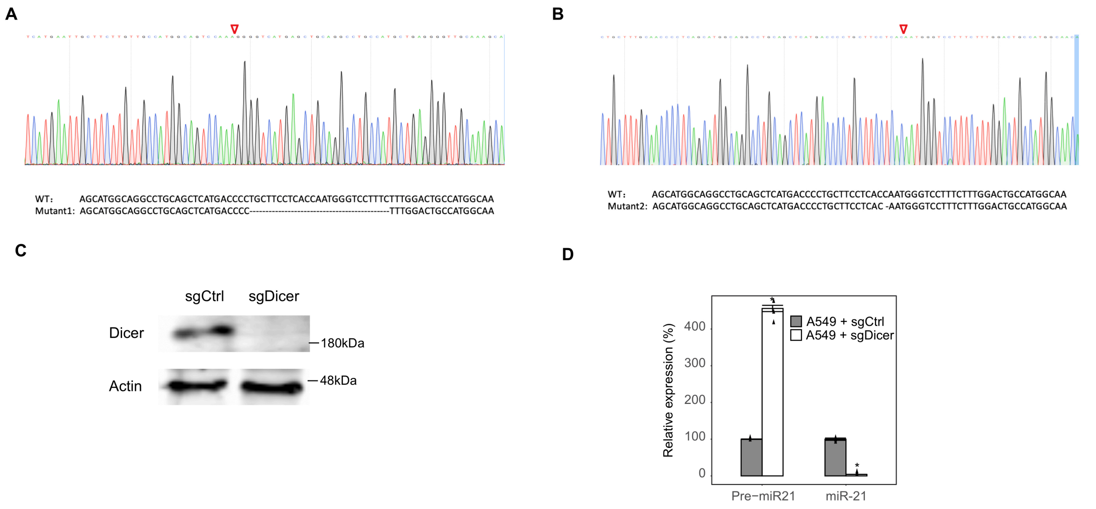

Supplementary Figure 5. **Knockdown of DICER1 abolishes ceRNA effects in A549 cells.**

(A) and (B) showing traces of genomic sequences of edited DICER1 coding region (upper panels) and corresponding primary sequences (lower panels) for the two alleles of a representative clone. Red triangles mark the mutation sites in sequencing traces. (C) Western blot showing the lack of DICER1 protein in edited A549 cells. (D) Barcharts showing the effects of DICER1 knockout on pre- and mature miRNA expression. n = 3; error bars indicate the means ± s.d. * $p < 0.01$, determined using a two-tailed Student's *t*-test.

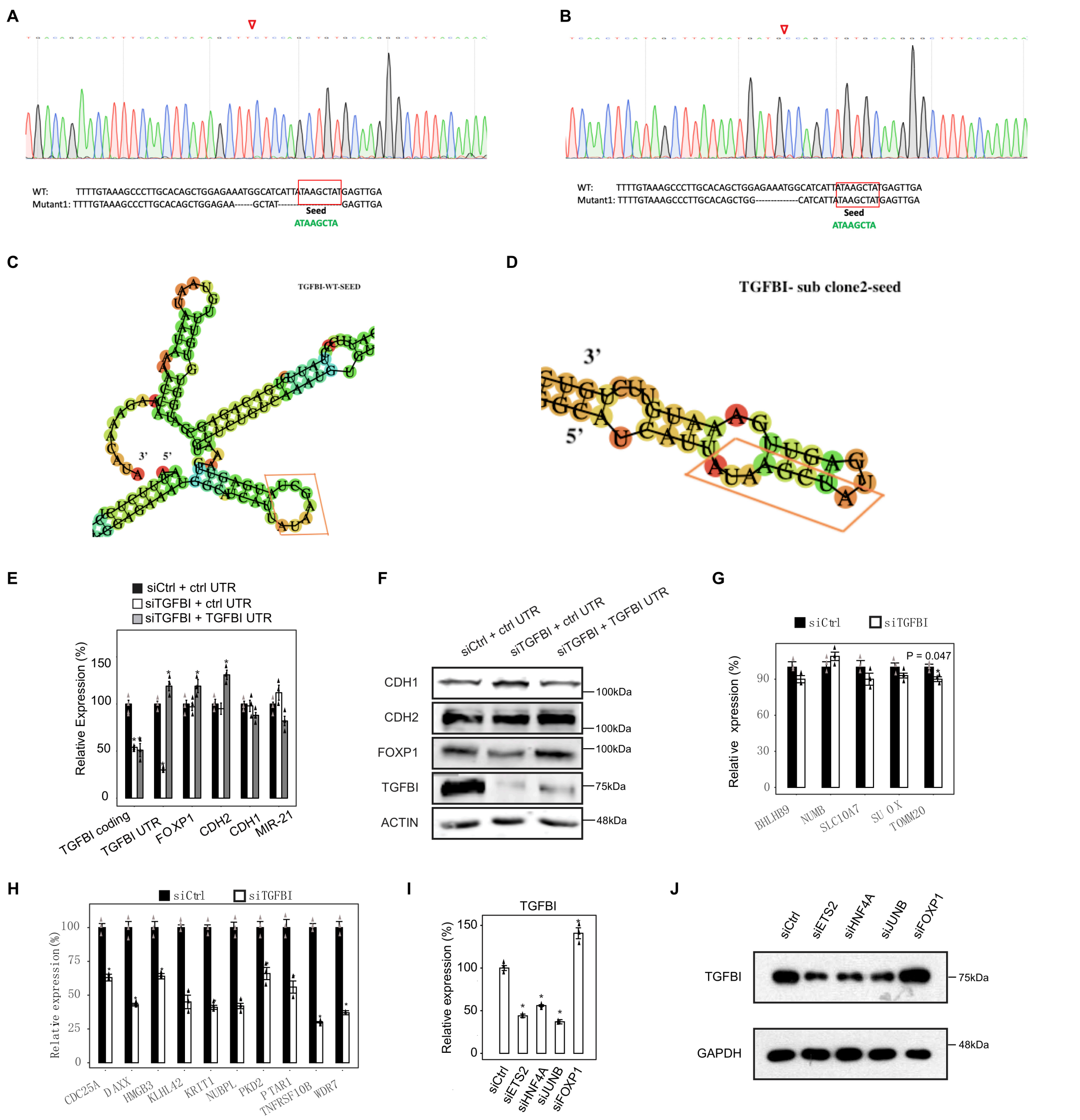

Supplementary Figure 6. **Mutating miR-21 binding site in TGFBI 3'UTR abolishes ceRNA effects in A549 cells.**

(A) and (B) showing traces of genomic sequence near the mutated miR-21 binding site in TGFBI 3'UTR (upper panels) and corresponding primary sequences (lower panels) for the two alleles of a representative clone. Red boxes highlight the mutated miR-21 binding sites. Red triangles mark the mutation sites in sequencing traces. (C) RNAduplex predicted secondary structure showing that the seed of miR-21 binding site in TGFBI 3'UTR is located in a loop region in wildtype cells. (D) RNAduplex predicted secondary structure showing that the mutated TGFBI 3'UTR as showed in (B) relocates the seed of miR-21 binding site to a stem region. (E) Graph showing the level of indicated RNAs in A549 cells during TGF-β-induced EMT subjected to indicated treatments using cells whose miR-21 binding site has been mutated with CRISPR/Cas9.

(F) Same as (E) for the immunoblotting analysis. (G) Graph showing the level of mRNAs for genes not targeted by miR-21 in A549 cells after knockdown of TGFBI. (H) same as (G) for known miR-21 target genes. (I) Graph showing the levels of TGFBI mRNA in A549 cells during TGF-β-induced EMT after silencing ETS2, HNF4A, JUNB or FOXP1 expression using specific siRNAs. (J) same as (I) for immunoblotting analysis. $n = 3$; error bars indicate the means ± s.d. * $p < 0.01$, determined using a two-tailed Student's *t*-test.

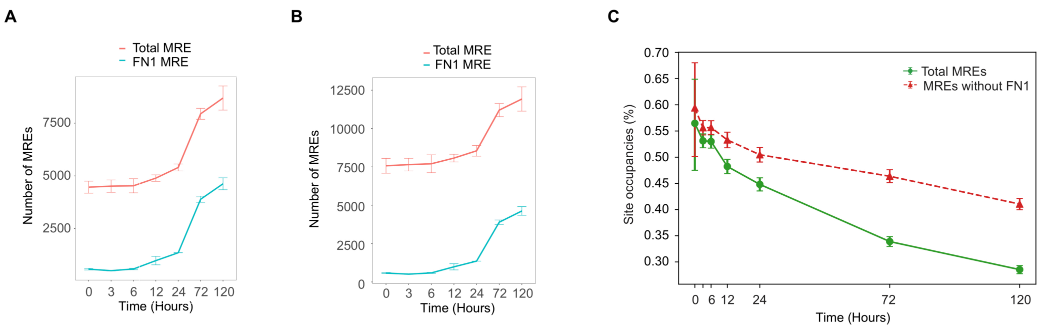

Supplementary Figure 7. **Dynamically induced mRNAs as ceRNA regulators of EMT in MCF10A cells.**

(A) Graph showing the number of miR-200c MREs extrapolated from microarray data during TGF-β-induced EMT in MCF10A cells using pictar-based predictions. (B) Graph showing the number of miR-200c MREs extrapolated from microarray data during TGF-β-induced EMT in MCF10A cells using targetScan-based predictions. (C) Graph showing the dynamics of modeled 8-mer miRNA binding site occupancy during TGF-β-induced EMT in MCF10A cells using targetScan-based predictions. n = 3; error bars indicate the means ± s.d.

A B

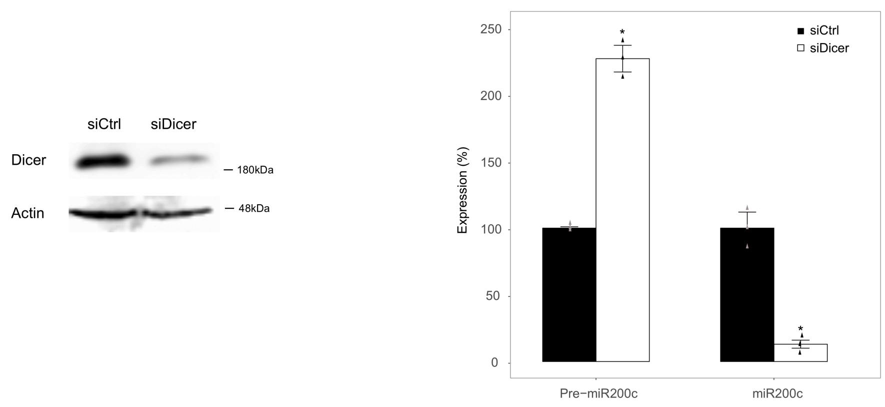

Supplementary Figure 8. **Knockdown of DICER1 abolish ceRNA effects in MCF10A cells.**

(A) Western blot showing the lack of DICER1 protein in treated MCF10A cells. (B) Barcharts showing the effects of DICER1 knockdown on pre- and mature miRNA expression. n = 3; error bars indicate the means ± s.d. * $p < 0.01$, determined using a two-tailed Student's *t*-test.

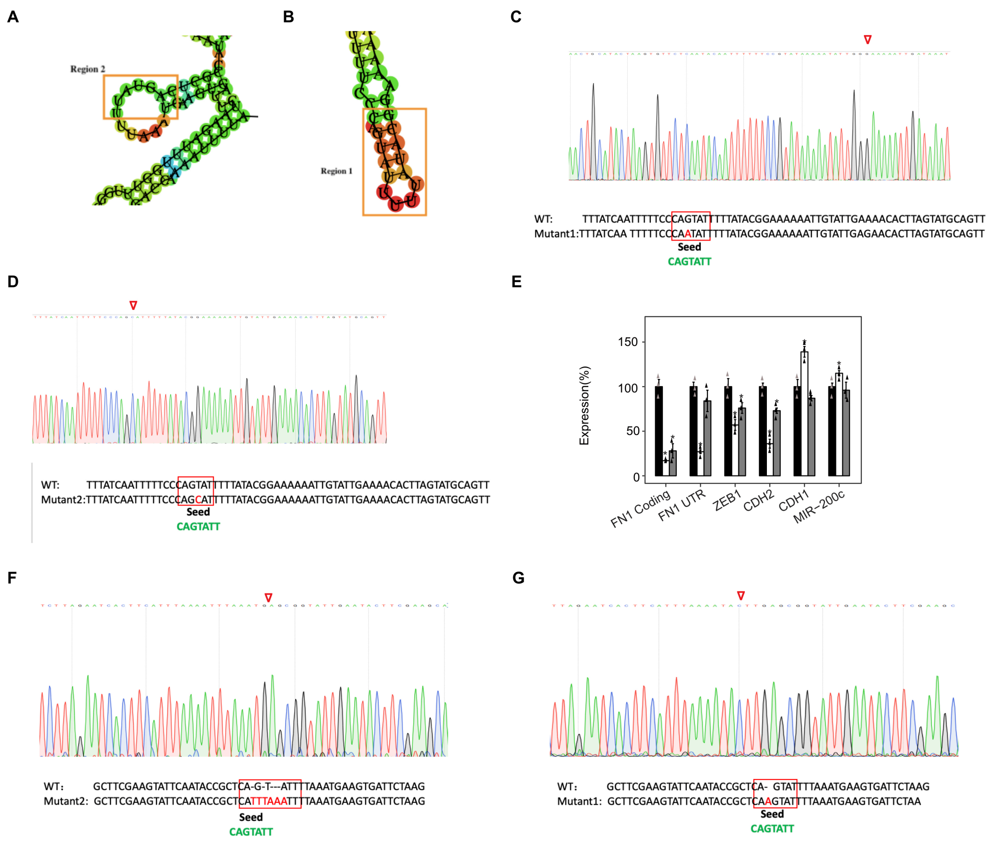

Supplementary Figure 9. **Mutating miR-200c binding site in FN1 3'UTR in MCF10A cells.**

(A) RNAduplex predicted secondary structure showing that the seed of the highly conserved miR-200c binding site in FN1 3'UTR is located in a loop region in wildtype cells. (B) RNAduplex predicted secondary structure showing that the seed of the poorly conserved miR-200c binding site in FN1 3'UTR is located in a stem region in wildtype cells. (C) and (D) showing traces of genomic sequence near the poorly conserved miR-200c binding site in FN1 3'UTR (upper panels) and corresponding primary sequences (lower panels) for the two alleles of a representative clone. Red boxes highlight the mutated miR-200c binding sites. Red triangles mark the mutation sites in sequencing traces. (E) Graph showing the level of indicated RNAs in MCF10A cells during TGF-β-induced EMT subjected to indicated treatments using cells whose poorly conserved miR-200c binding site has been mutated with CRISPR/Cas9. (F) and (G) same as (C) and (D) for the highly conserved miR-200c binding site in FN1 3'UTR. n = 3; error bars indicate the means ± s.d. * $p < 0.01$, determined using a two-tailed Student's *t*-test.

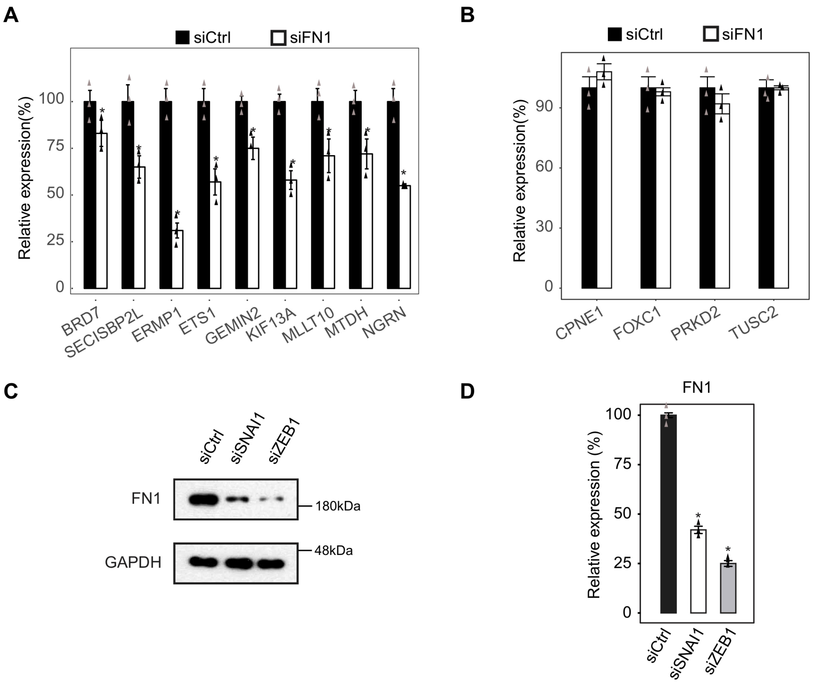

Supplementary Figure 10. **Mutating miR-200c binding site in FN1 3'UTR abolishes ceRNA effects in A549 cells.**

(A) Graph showing the level of RNAs for genes targeted by miR-200c in MCF10A cells after knockdown of FN1. (B) same as (A) for genes not targeted by miR-200c. (C) Immunoblotting analysis showing the abundance of FN1 protein in MCF10A cells during TGF-β-induced EMT after silencing SNAI1 or ZEB1 expression using specific siRNAs. (D) Same as (C) for FN1 mRNA. n = 3; error bars indicate the means ± s.d. * $p < 0.01$, determined using a two-tailed Student's *t*-test.

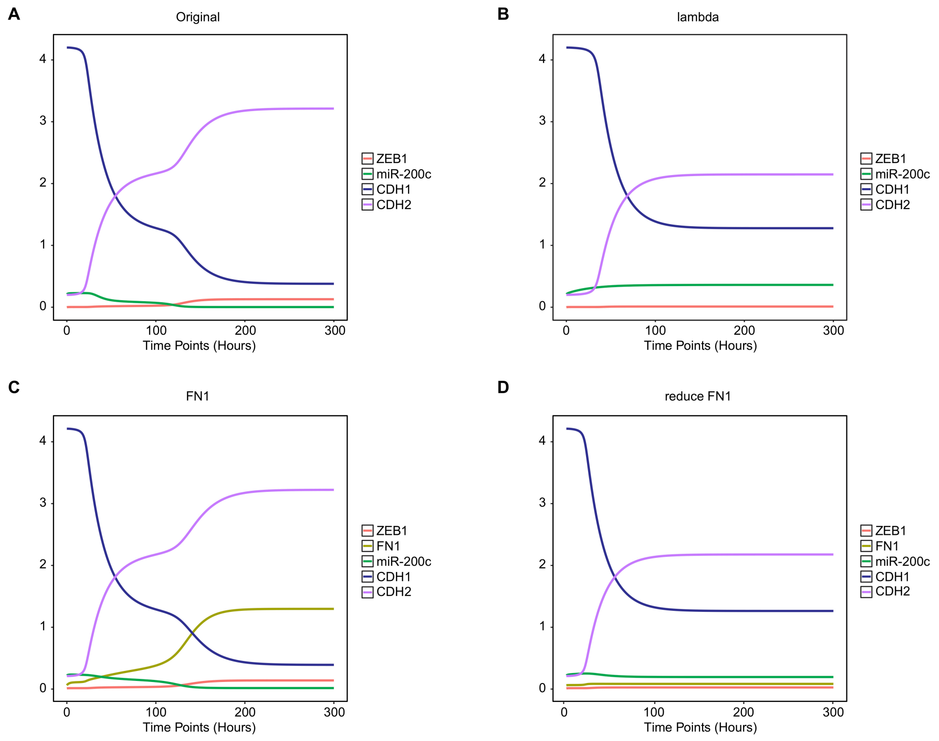

Supplementary Figure 11. **Simulated time-course dynamics of EMT genes.**

(A) Graph showing the time-course expression of key EMT genes during a 300h simulation using the model originally developed by Zhang et al. (B) Same as (A) but with lambda(1-5) increased from 0.5 to 0.9 to increase the half-life of miR-200c. (C) Graph showing the time-course expression of key EMT genes during a 300h simulation using the model incorporating FN1. (D) Same as (C) but with the FN1 degradation rate constant increased to 1 to simulate FN1 knockdown.

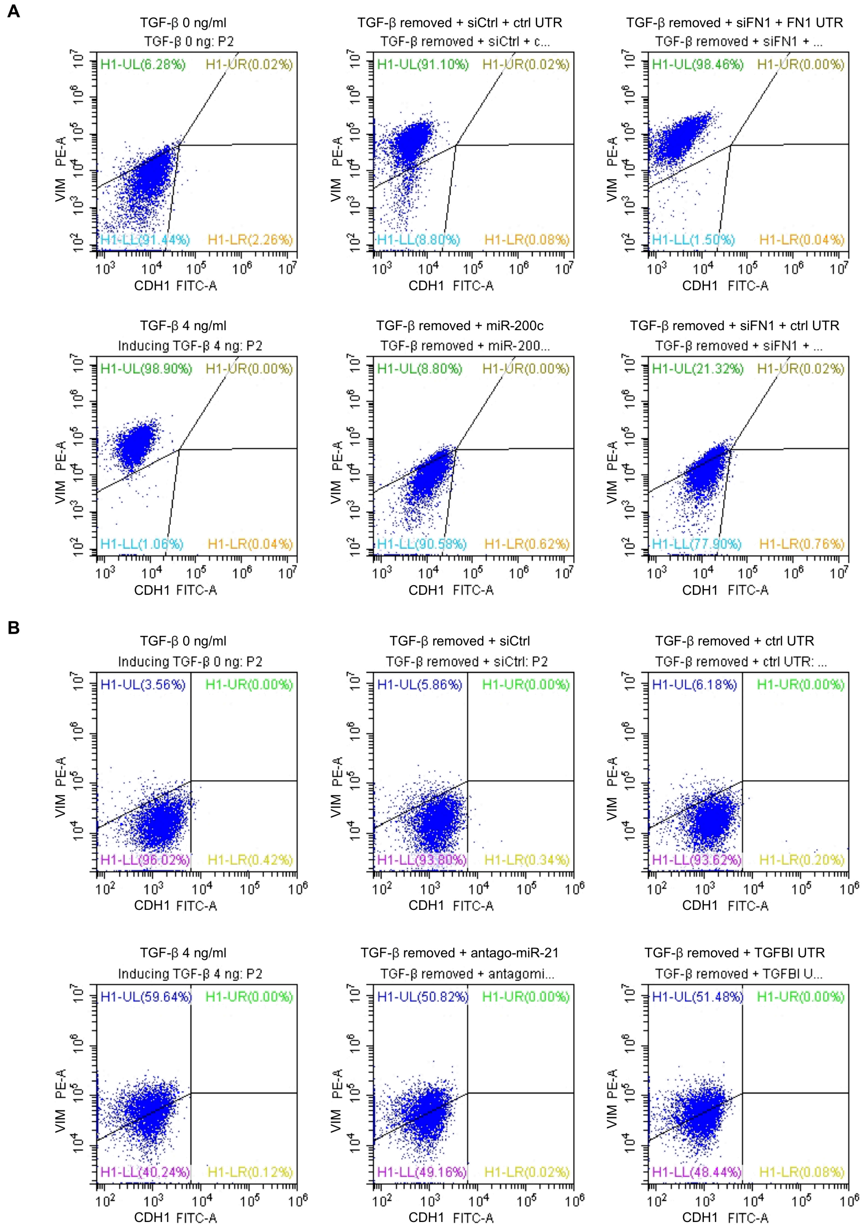

Supplementary Figure 12. **The stoichiometry between ceRNA and miRNA determines EMT reversibility.**

(A) MCF10A cells were treated with 0 or 4 ng/ml TGF-β for 7 days to induce EMT, followed by the indicated treatment for 3 days. The abundance of E-cadherin and Vimentin was analyzed via flow cytometry. The pictures are representative of three biological replicates. (B) Same as (A) for A549 cells.

Supplementary Tables.

Supplementary Table 1. Representative TPM of miR-21 target mRNAs during EMT in A549 cells.*

Symbol	Isoform	0h	6h	12h	24h	36h	48h	72h	96h
TGFBI	ENST00000442011	288.48	748.27	1621.84	2721.68	3237.3	3215.27	2618.61	1409.31
VCL	ENST00000372755	71.95	76.95	135.5	147.15	100.32	88.37	96.67	73.64
TGFBI	ENST00000506699	8.03	49.29	64.87	99.12	105.41	80.36	59.71	22.94
RTN4	ENST00000317610	59.61	50.22	64.18	63.05	58.62	50.88	56.7	55.95
JAG1	ENST00000254958	12.43	17.87	28.31	43.23	46.99	41.68	55.62	38.55
MPRIP	ENST00000341712	31.49	32.11	34.79	41.31	29.51	15.41	18.23	13.1
RAB11A	ENST00000564910	36.62	34.39	35.67	38.19	33.09	34.41	36.72	29.34
TGFB2	ENST00000366930	22.27	24.04	28.17	34.7	31.43	29.33	25.49	16.73
SCRN1	ENST00000242059	36.55	29.34	30.19	28.16	27.42	25.93	24.73	21.02
TGFBI	ENST00000504411	3.66	11.43	19.47	25.77	27.28	22.59	10.99	4.14
BMPR2	ENST00000374580	7.17	14.26	18.88	23.74	21.26	11.38	14.13	6.09
STAG2	ENST00000371157	19.46	14.51	20.31	20.33	19.99	19.29	15.66	13.12
UBE2D3	ENST00000394803	19.31	23.89	23.6	19.26	13.71	17.86	14.45	14.92
RAB11A	ENST00000261890	20.22	16.8	19.07	18.71	19.59	19.74	19.46	14.5
MPRIP	ENST00000395811	17.13	8.26	12.12	18.68	16.06	9.11	10.27	5.71
RAB11A	ENST00000569896	13.92	15.79	15.3	18.45	17.53	18.2	15.2	15.14
KPNA4	ENST00000334256	17.23	17.72	18.81	16.82	13.48	12.01	12.6	9.82
SOCS6	ENST00000397942	16.15	11.12	13.42	15.99	23.04	30.64	26.43	21.1
MSH2	ENST00000233146	26.22	22.92	18.05	15.81	15.41	16.61	17.61	14.48
LANCL1	ENST00000450366	18.22	13.3	12.39	13.4	13.74	15.23	13.83	14.85
SMAD7	ENST00000262158	5.14	11.44	11.67	12.77	10.47	7.17	9.98	5.78
YAP1	ENST00000282441	10.67	7.26	9.98	10.95	8.96	10.1	10.71	9.36
UBE2D3	ENST00000394801	11.37	12.47	10.82	10.63	11.56	8.82	12.47	11.92
HIPK3	ENST00000303296	10.03	9.62	6.75	10.44	6.37	10.9	9.97	4.96

* Only isoforms with TPM > 10 at 24h into EMT are shown. Data is taken from replicate 1 of GSE69667.

Supplementary Table 2. MREs for miR-21 derived from TargetScan during EMT in A549 cells.*

Time	# MREs from TGFBI	Total # of MREs	# of increased MREs from TGFBI comparing to 0h	Total # of increased MREs comparing to 0h	Percentage of increased MREs contributed by TGFBI
0h	498	1,554	0	0	NA
6h	872	1,716	373	161	230%
12h	2,201	3,577	1,702	2,022	84%
24h	2,842	4,070	2,343	2,516	93%
36h	3,271	4,469	2,772	2,915	95%
48h	2,587	3,447	2,088	1,892	110%
72h	2,587	3,723	2,088	2,168	96%
96h	1,936	3,057	1,438	1,502	95%

* MREs were before scaling with absolute qPCR data.

Supplementary Table 3. Representative TPM of miR-200c target mRNAs during EMT in MCF10A cells.*

Symbol	Isoform	0h	3h	6h	12h	24h	72h	120h
FN1	ENST00000336916	218.15	194.4696674	222.1218974	388.1095348	517.3348997	1490.119701	1744.526457
FN1	ENST00000357867	217.48	193.8723964	221.4396986	386.9175413	515.7460187	1485.543124	1739.168526
FN1	ENST00000456923	206.22	183.8346771	209.9746857	366.8849336	489.0433327	1408.629313	1649.123291
NPM1	ENST00000517671	1236.67	1306.725747	1246.622555	1280.029453	1234.432937	1257.135243	1411.762355
FN1	ENST00000421182	111.48	99.37876932	113.509737	198.3334904	264.3708211	761.4877115	891.4958031
FN1	ENST00000443816	100.64	89.71545877	102.4723711	179.0481026	238.6641499	687.4427995	804.8092718
FN1	ENST00000323926	37.84	33.73244197	38.52895988	67.32094796	89.73620264	258.4741209	302.6031682
NPM1	ENST00000351986	193.56	204.5249223	195.117745	200.3464958	193.2098614	196.7631604	220.9649473
RHOA	ENST00000418115	166.59	170.4329788	161.6843865	180.5195562	163.8056198	177.9292789	195.4089932
YWHAQ	ENST00000238081	143.39	162.9973397	166.7990172	168.012319	162.0424524	168.1526637	190.0838966
SERF2	ENST00000409614	150.19	141.515457	153.0885569	131.8141477	131.6727301	151.8285129	153.6924672
HSPA9	ENST00000297185	150.33	152.4771205	152.988219	148.5528113	157.6793856	145.5471835	169.809349
DDIT4	ENST00000307365	172.28	141.2570047	132.1644885	136.3792126	153.148988	138.1825242	79.99864564
FSTL1	ENST00000295633	45.06	51.53215074	66.64270324	78.45239938	90.06475148	123.5215759	139.4024721
NPM1	ENST00000296930	116.74	123.3531692	117.6795079	120.8330746	116.5288243	118.6718917	133.2684849
GDI2	ENST00000380191	118.51	113.4256029	122.4551297	115.3985197	109.5783983	107.3015832	120.5353254
HNRNPK	ENST00000376281	97.29	123.0332702	97.88238055	88.60202383	95.71614136	97.94893847	102.0991836
ETF1	ENST00000360541	87.68	88.39490636	82.10596601	80.48847112	84.8576093	86.61150113	95.89569855
HNRNPK	ENST00000360384	81.4	102.9387213	81.89562932	74.13099743	80.08319361	81.95131659	85.4237182
FSTL1	ENST00000424703	29.22	33.41698723	43.21570769	50.87392609	58.40417306	80.09987676	90.39814101
MSN	ENST00000360270	61.04	71.61872518	76.88584587	73.3466566	66.7003055	79.1416521	75.81215871
YWHAB	ENST00000353703	67.12	67.96575468	79.73754397	69.97046353	71.86891001	75.81649932	104.2279166
SCD	ENST00000370355	69.6	75.54895952	73.47699757	69.63295152	75.07596693	75.49159188	60.76853911
HNRNPH1	ENST00000442819	67.5	69.62688099	65.77859133	66.20859737	80.10018398	73.95756061	84.43658565
SERINC1	ENST00000339697	57.82	53.86475714	62.12141695	58.48621947	64.0946661	73.87149167	80.16246806
HAS2	ENST00000303924	11.01	8.799935081	10.08342441	14.62964421	22.39759645	69.58670458	67.43490269
PRKAR1A	ENST00000358598	57.08	62.47690424	68.11480071	64.32878981	78.43650994	69.4510315	93.59488329
DHX9	ENST00000367549	62.67	60.31315929	58.02458044	60.25724736	60.06180501	61.36469369	62.48261492
AHNAK	ENST00000378024	64.33	50.31201288	41.96601294	45.5871644	64.7337667	60.46195277	48.89967866
FERMT2	ENST00000341590	34.81	39.05346554	39.39222348	44.93233922	55.91425309	60.00271	65.19608486
HNRNPU	ENST00000440865	83.58	72.7714877	58.41560554	61.96430465	82.70610042	59.3497176	37.55328565
MFAP5	ENST00000359478	1.92	1.749122893	10.745876	1.362589197	1.440498374	54.1417911	0.535907629
RAC1	ENST00000348035	56.39	50.52308793	52.15738695	54.91387835	46.61281219	53.35004323	57.34147416
SSR3	ENST00000265044	42.44	47.31662733	43.07088867	42.58786893	36.57239432	51.50046239	54.43564608
APLP2	ENST00000278756	50	42.67900914	47.88043756	51.68234377	55.36853395	50.88366248	45.5221682
PLS3	ENST00000355899	52.13	55.24744754	57.00376948	55.55695374	59.33643325	50.67590928	56.8522669
SRSF1	ENST00000582730	59.45	54.49509273	47.97438992	55.56535187	58.22428338	50.03156926	56.2999231
YWHAG	ENST00000307630	52.13	46.80480648	44.63275078	50.45720699	58.91846177	49.40816013	39.23687753
SERF2	ENST00000339624	48.2	45.41610646	49.13022468	42.30269603	42.25731135	48.72584275	49.32403569
LAMC1	ENST00000258341	50.89	49.74757564	47.05823871	43.66267328	53.13061409	47.78911857	44.53720429
LAMC1	ENST00000258341	50.89	49.74757564	47.05823871	43.66267328	53.13061409	47.78911857	44.53720429
RAP1B	ENST00000393436	43.68	43.70123824	45.51455976	42.12859086	40.77725705	46.97169	55.35045206
SHC1	ENST00000368453	38.75	42.98141461	42.22759247	36.42219795	43.59413103	46.50348379	42.40591604
YME1L1	ENST00000376016	38.5	39.44502162	38.27567762	37.02989186	39.82341519	45.84909843	39.15577724
ANLN	ENST00000265748	36.34	36.57354212	39.05144624	40.69339151	45.03275046	43.67700781	59.92151983
PTPN11	ENST00000351677	36.14	39.7057089	40.90019355	34.52618887	37.13749112	41.62203628	42.17728724
SHC1	ENST00000448116	32.2	35.71616905	35.08976716	30.26567159	36.22531662	38.64289492	35.2379483
FRMD6	ENST00000395718	25.58	29.64869257	29.87433362	27.99160091	29.02920235	37.93865501	48.03260161
PCNP	ENST00000265260	33.99	34.13669932	35.11046938	30.34306699	31.98796271	37.69473224	37.1741179

* Top 50 isoforms at 72h into EMT are shown.

Supplementary Table 4. MREs for miR-200c derived from TargetScan during EMT in MCF10A cells.*

Time	# MREs from FN1	Total # of MREs	# of increased MREs from FN1 comparing to 0h	Total # of increased MREs comparing to 0h	Percentage of increased MREs contributed by FN1
0h	893	11678	0	0	NA
3h	796	11828	-97	149	-64%
6h	909	11869	16	190	8%
12h	1589	12572	696	894	77%
24h	2119	13243	1225	1564	78%
72h	6104	17379	5210	5700	91%
120h	7146	18388	6252	6709	93%

* MREs were before scaling with absolute qPCR data.

Supplementary Table 5. MiRNA expression in MCF10A cells (GSE50064).

	normalized count
miR-378a-3p	5,886,078
miR-378c	5,875,199
miR-378d	5,868,998
let-7a-5p	3,087,464
let-7c	2,929,684
let-7f-5p	1,457,612
miR-103a-3p	559,644
miR-103b	559,644
miR-107	541,327
miR-21-5p	486,038
miR-27a-3p	478,586
miR-27b-3p	477,975
miR-100-5p	221,489
miR-200c-3p	211,178

Supplementary Table 6. Conserved miRNA binding sites in ZEB1 3'UTR as predicted by targetScan 7.2.

**miRNA family**	**Total**	**8mer**	**7mer-m8**	**7mer-A1**
miR-200bc-3p/429	5	**3**	**2**	0
miR-141-3p/200a-3p	3	**1**	**2**	0
miR-183-5p.2	2	0	**1**	**1**
miR-139-5p	2	**1**	**1**	0
miR-101-3p.1	2	**1**	0	**1**
miR-101-3p.2	2	0	**2**	0
miR-144-3p	2	**1**	0	**1**
miR-124-3p.2/506-3p	1	0	0	**1**
miR-96-5p/1271-5p	1	0	**1**	0
miR-205-5p	1	**1**	0	0
miR-183-5p.1	1	0	**1**	0
miR-128-3p	1	0	**1**	0
miR-150-5p	1	**1**	0	0
miR-223-3p	1	0	0	**1**
miR-130-3p/301-3p/454-3p	1	0	0	**1**
miR-199-3p	1	0	**1**	0
miR-142-3p.1	1	0	0	**1**
miR-455-3p.1	1	0	**1**	0
miR-199-5p	1	0	**1**	0
miR-143-3p	1	0	0	**1**
miR-181-5p	1	0	0	**1**
miR-23-3p	1	0	**1**	0
miR-194-5p	1	0	0	**1**
miR-216a-5p	1	0	**1**	0
miR-203a-3p.2	1	0	**1**	0
miR-203a-3p.1	1	**1**	0	0
miR-204-5p/211-5p	1	**1**	0	0
miR-802	1	0	0	**1**

Supplementary Table 7. Parameters and variables adjusted or added in the EMT model incorporating FN1.

**Parameter or variable**	**Description**	**Original Value**	**Adjusted/added value**
Lamda1-5	Recycling ratio of miR-200 from degraded miR-200-ZEB1 complex	0.5	0.9
Lamdafn1	Recycling ratio of miR-200 from degraded miR-200-FN1 complex	NA	0.86
$k0_{fn1}$	Transcription rate constant of Fn1	NA	0.05 μM/hr
kd_{fn1}	Degradation rate constant of free Fn1	NA	0.08/hr
kd_{fR1}	Degradation rate constant of Fn1-miR-200 complex	NA	0.9/hr
K_{fn1}	Binding rate constant of miR-200 to Fn1	NA	10 /μM
ks_{fn1}	Transcription rate constant of SNAI1 on Fn1	NA	0.02 μM/hr
kz_{fn1}	Transcription rate constant of ZEB1 on Fn1	NA	0.04 μM/hr
Js_{fn1}	Michaelis constant of SNAI1 regulation of Fn1	NA	0.2 μM
Jz_{fn1}	Michaelis constant of ZEB1 regulation of Fn1	NA	0.5 μM
$[Fn1]_t$	Initial concentration of Fn1 transcripts	NA	0.05 μM

*NA indicates that the parameter or variable is not in the EMT model of Zhang et al.

Supplementary Note. ODEs describing the EMT model incorporating FN1.

```
# The model was based on the CBS EMT model developed at Dr. Jianhua Xing’s group.
# Please refer to Zhang, J.Y. et al. TGF-beta-induced epithelial-to-mesenchymal
# transition proceeds through stepwise activation of multiple feedback loops. Sci
# Signal 7 (2014).

p TGF0=0
p Ks=100, lamdas=0.5

#Time scale for mRNA and miRNA binding and unbinding, which is assumed very fast.

p Timescale=1000
TGFt=TGF+TGF0
miR34=miR34t-SR1
snail=snailt-SR1
snailt'= k0_snail + k_snail*(TGFt/J_snail0)^2 /(1+(TGFt/J_snail0)^2 )/(1+SNAIL/J_snail1)-
kd_snail*snail-kd_SR1*SR1

SNAIL'= k_SNAIL*snail-kd_SNAIL*SNAIL

miR34t'= k0_34 + k_34/( 1 + (SNAIL/J1_34)^2+ (ZEB/J2_34)^2)- kd_34*miR34-
kd_SR1*SR1+lamdas*kd_SR1*SR1

SR1'=Timescale*(Ks*snail*miR34-SR1)

 p k0_snail=.0006, k_snail=0.05, J_snail0=.62, J_snail1=.67, kd_snail=0.09, kd_SR1=0.9 ,

p k_SNAIL=17,  kd_SNAIL=1.66,

p k0_34=0.0012, k_34=0.012, J1_34=0.15, J2_34=0.36,  kd_34=0.035,


#####################################################################################
###

p K1=10, K2=10, K3=10, K4=10, K5=10

p lamda1=0.9, lamda2=0.9, lamda3=0.9, lamda4=0.9, lamda5=0.9

p dk_ZR1=0.9, dk_ZR2=0.9, dk_ZR3=0.9, dk_ZR4=0.9, dk_ZR5=0.9

 zebt'= k0_zeb + k_zeb*(SNAIL/J_zeb)^2 /( 1 + (SNAIL/J_zeb)^2 )- kd_zeb*zeb-dk_ZR1*5*ZR1-
dk_ZR2*10*ZR2-dk_ZR3*10*ZR3-dk_ZR4*5*ZR4-dk_ZR5*ZR5

ZEB'= k_ZEB*zeb-kd_ZEB*ZEB


ZR1'=Timescale*(K1*miR200*zeb-ZR1)

ZR2'=Timescale*(K2*miR200*ZR1-ZR2)

ZR3'=Timescale*(K3*miR200*ZR2-ZR3)

ZR4'=Timescale*(K4*miR200*ZR3-ZR4)

ZR5'=Timescale*(K5*miR200*ZR4-ZR5)
```

```
zeb=zebt-(5*ZR1+10*ZR2+10*ZR3+5*ZR4+ZR5)

 p k0_zeb=.003, k_zeb=0.06,  J_zeb=3.5,  kd_zeb=0.09,

p k_ZEB=17, J_ZEB=0.06,  kd_ZEB=1.66,

p k0_200=0.0002, k_200=0.02, J1_200=3.25, J2_200=0.2, kd_200=0.035,


#####################################
# FN1 related parameters and ODEs#
#####################################

p k0_fn1=0.05, kd_fn1=0.08, kd_fR1=0.9

p Kfn1=10

p ks_fn=0.02, kz_fn=0.04, Js_fn1=0.2, Jz_fn1=0.5

p lamdafn1=0.86

fnt'=k0_fn1+ks_fn*(SNAIL/Js_fn1)^2/(1+(SNAIL/Js_fn1)^2)+kz_fn*(ZEB/Jz_fn1)^2/(1+(ZEB/J
z_fn1)^2)-kd_fn1*fn1-kd_fR1*fR1

fn1=fnt-fR1

fR1'=Timescale*(Kfn1*miR200*fn1-fR1)

miR200t'= k0_200 + k_200/( 1 + (SNAIL/J1_200)^3 + (ZEB/J2_200)^2)-kd_200*miR200 -
dk_ZR1*5*ZR1-dk_ZR2*2*10*ZR2-dk_ZR3*3*10*ZR3-dk_ZR4*4*5*ZR4-
dk_ZR5*5*ZR5+lamda1*dk_ZR1*5*ZR1+lamda2*dk_ZR2*2*10*ZR2+lamda3*dk_ZR3*3*10*
ZR3+lamda4*dk_ZR4*4*5*ZR4+lamda5*dk_ZR5*5*ZR5-                     kd_tgfR*tgfR+
lamdatgfR*kd_tgfR*tgfR-kd_fR1*fR1+lamdafn1*kd_fR1*fR1

miR200=miR200t-(5*ZR1+2*10*ZR2+3*10*ZR3+4*5*ZR4+5*ZR5)-tgfR-fR1

#########################################################################################
#####################

 p KTGF=20, TGF_flg=0

p k_tgf=0.05, kd_tgf=0.09, kd_tgfR=0.9,lamdatgfR=0.8

p k_TGF=1.6, kd_TGF=1,

tgft' = k_tgf - kd_tgf*tgf- kd_tgfR*tgfR

TGF' = k_TGF*tgf - kd_TGF*TGF

 tgf=tgft-tgfR

tgfR'=Timescale*(TGF_flg+KTGF*miR200*tgf-tgfR)
```

```
#####################################################################################
#####################

p k_ecad0=0.01, k_ecad1=0.15, k_ecad2=.05, J_ecad1=.2, J_ecad2=0.5,kd_ecad=.05,

p k_ncad0=0.01, k_ncad1=0.1, k_ncad2=.06, J_ncad1=.2, J_ncad2=0.5,kd_ncad=.05,

E_cadherin'=k_ecad0+k_ecad1/((SNAIL/J_ecad1)^2              +1)+k_ecad2/((ZEB/J_ecad2)^2+1)-
kd_ecad*E_cadherin

N_cadherin'=k_ncad0+k_ncad1*((SNAIL/J_ncad1)^2)/((SNAIL/J_ncad1)^2+1)+k_ncad2*((ZEB/
J_ncad2)^2)/((ZEB/J_ncad2)^2+1)-kd_ncad*N_cadherin

 init snailt=0,SNAIL=0,miR34t=0.37

init zebt=0,ZEB=0,miR200t=0.21,ZR1=0,ZR2=0,ZR3=0,ZR4=0,ZR5=0,ZR6=0

init tgfmt=0.07,TGF=0.03,tgfR=0.05,E_cadherin=4.2,N_cadherin=0.2

init fnt=0.05

done
```
